# Development of Graves' Disease After SARS-CoV-2 mRNA Vaccination: A Case Report and Literature Review

**DOI:** 10.3389/fpubh.2021.778964

**Published:** 2021-11-23

**Authors:** David Tak Wai Lui, Ka Kui Lee, Chi Ho Lee, Alan Chun Hong Lee, Ivan Fan Ngai Hung, Kathryn Choon Beng Tan

**Affiliations:** Department of Medicine, The University of Hong Kong, Queen Mary Hospital, Hong Kong, Hong Kong SAR, China

**Keywords:** COVID-19, SARS-CoV-2, COVID-19 vaccines, Graves' disease, thyroiditis, autoimmunity

## Abstract

**Background:** Mounting evidence has revealed the interrelationship between thyroid and severe acute respiratory syndrome coronavirus 2 (SARS-CoV-2) to explain the thyroid dysfunction and autoimmune thyroid disorders observed after coronavirus disease 2019 (COVID-19). There are limited reports of thyroid dysfunction after SARS-CoV-2 vaccination.

**Methods:** We report a case of a 40-year-old Chinese woman who developed Graves' disease after BNT162b2 mRNA vaccine. A search of PubMed and Embase databases from 1 September 2019 to 31 August 2021 was performed using the following keywords: “COVID,” “vaccine,” “thyroid,” “thyroiditis,” and “Graves.”

**Results:** A 40-year-old Chinese woman who had 8-year history of hypothyroidism requiring thyroxine replacement. Her anti-thyroid peroxidase and anti-thyroglobulin antibodies were negative at diagnosis. She received her first and second doses of BNT162b2 mRNA vaccine on 6 April and 1 May 2021, respectively. She developed thyrotoxicosis and was diagnosed to have Graves' disease 5 weeks after the second dose of vaccine, with positive thyroid stimulating immunoglobulin level, diffuse goiter with hypervascularity on thyroid ultrasonography and diffusely increased thyroid uptake on technetium thyroid scan. Both anti-thyroid peroxidase and anti-thyroglobulin antibodies became positive. She was treated with carbimazole. Literature search revealed four cases of Graves' disease after SARS-CoV-2 vaccination, all after mRNA vaccines; and nine cases of subacute thyroiditis, after different types of SARS-CoV-2 vaccines.

**Conclusion:** Our case represents the fifth in the literature of Graves' disease after SARS-CoV-2 vaccination, with an unusual presentation on a longstanding history of hypothyroidism. Clinicians should remain vigilant about potential thyroid dysfunction after SARS-CoV-2 vaccination in the current pandemic.

## Introduction

Hopefully vaccination against severe acute respiratory syndrome coronavirus 2 (SARS-CoV-2) will bring an end to the current intense fight against the coronavirus disease 2019 (COVID-19) pandemic. Following reports of COVID-related thyroiditis and autoimmune thyroid disorders such as Graves' disease (1), there are concerns that SARS-CoV-2 vaccination may also be associated with thyroid dysfunction.

Indeed, there are several case reports of thyroiditis after SARS-CoV-2 vaccination. These events of thyroiditis occurred after mRNA vaccines (2, 3), inactivated vaccines (3–6) and adenovirus-vectored SARS-CoV-2 vaccines (7). In contrast, reports of Graves' disease occurring after SARS-CoV-2 vaccines are restricted to after mRNA vaccines (8, 9).

Here we report a 40-year-old Chinese woman who developed Graves' disease after SARS-CoV-2 mRNA vaccination, on a background of longstanding hypothyroidism stable on thyroxine replacement. We also take this opportunity to review the currently available reports of thyroid dysfunction after SARS-CoV-2 vaccination.

## Materials and Methods

Written informed consent was obtained from the patient for publication of this case report and any accompanying images. We performed a literature search of PubMed and Embase databases regarding thyroid dysfunction after SARS-CoV-2 vaccination, from 1 September 2019 to 31 August 2021, using the following keywords: “COVID,” “vaccine,” “thyroid,” “thyroiditis,” and “Graves.”

## Results

### Case Presentation

A 32-year-old Chinese woman, with good past health and no family history of thyroid disorders, presented in September 2013 with cold intolerance and lethargy. Thyroid function test showed subclinical hypothyroidism: thyroid stimulating hormone (TSH) level was 5.30 mIU/L (reference range: 0.27–4.20) and free thyroxine (fT4) level was 13.9 pmol/L (reference range: 12.0–22.0), similar on repeat. Her anti-thyroid peroxidase (anti-TPO) and anti-thyroglobulin (anti-Tg) were negative (anti-TPO <5.0 IU/mL, reference range <34.0; anti-TPO <10 IU/mL, reference range <115). She was initially given an intermediate dose of thyroxine 50 microgram daily (i.e., 350 microgram weekly; her body weight was 50 kg), and stabilized on a thyroxine dose of 50 microgram for 5 days per week and 100 microgram daily for 2 days per week, totally 450 microgram per week. Subsequent thyroid function remained normal with this dosage of thyroxine. Her symptoms also improved with thyroxine replacement. Her serial thyroid function test results are listed in Table 1. Routine follow-up in July 2020 showed normal TSH and fT4. She remained clinically euthyroid.

**Table 1:** Serial thyroid function tests and anti-thyroid antibody titers in our reported case.

**Date**	**TSH (mIU/L)**	**RR**	**fT4 (pmol/L)**	**RR**	**fT3 (pmol/L)**	**RR**
**At diagnosis**						
7 Jan 2013	5.30	0.27–4.20	13.9	12.0–22.0	3.58	3.10–6.80
**Stable on thyroxine replacement**						
12 Oct 2017	1.03	0.35–4.94	14.82	9.01–19.05		
17 Apr 2018	3.74	0.35–4.94	16.60	9.01–19.05		
23 Aug 2018	0.94	0.35–4.94	14.69	9.01–19.05		
26 Feb 2019	1.67	0.35–4.94	12.41	9.01–19.05		
11 Sep 2019	1.35	0.35–4.94	15.23	9.01–19.05		
2 Jul 2020	0.86	0.35–4.94	14.35	9.01–19.05		
**After SARS-CoV-2 vaccination**						
8 Jun 2021	<0.02	0.47–4.68	66.6	10.0–28.2	30.50	4.26–8.10
**Started carbimazole**						
23 Jun 2021			23.70	9.01–19.05	11.05	2.63–5.70
21 Jul 2021			16.94	9.01–19.05	6.01	2.63–5.70

*TSH, thyroid-stimulating hormone; fT4, free thyroxine; fT3, free triiodothyronine; RR, reference range*.

In the COVID-19 pandemic, now 40 years of age, she has not been infected with COVID-19. She received her first and second doses of BNT162b2 mRNA vaccine (Comirnaty) on 6 April and 1 May 2021, respectively. Subsequently, she presented with palpitation and was noted to have sinus tachycardia upon visit to private practitioner on 8 June 2021.

**Figure 1:**
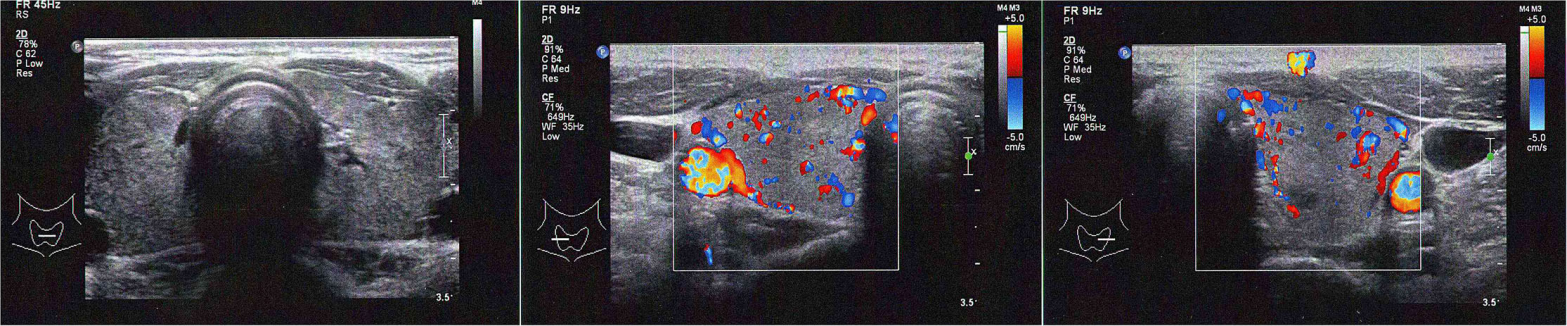
Technetium thyroid scan at the diagnosis of Graves' disease of our patient.

**Figure 2:**
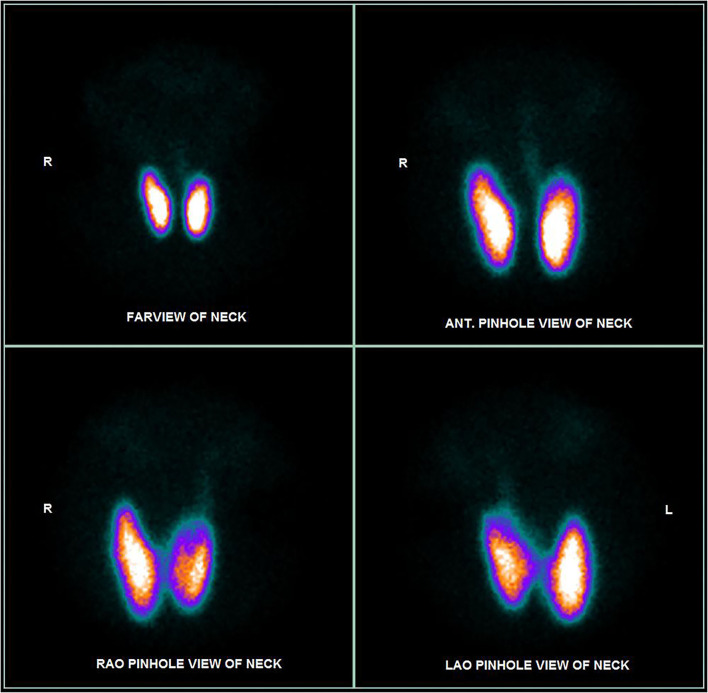
Thyroid ultrasonography at the diagnosis of Graves' disease of our patient (left panel: both lobes without Doppler; mid panel: right lobe with Doppler; right panel: left lobe with Doppler).

Blood tests revealed a picture of thyrotoxicosis: TSH <0.02 mIU/L (reference range: 0.47–4.68), fT4 66.6 pmol/L (reference range: 10.0–28.2) and fT3 30.50 pmol/L (reference range: 4.26–8.10). Physical examination revealed a moderate diffuse goiter with thyroid bruit. She did not have signs of Graves' orbitopathy or dermopathy. Her thyroid stimulating immunoglobulin (TSI) level was 420% (reference range: <140% baseline). Interestingly, both her anti-TPO and anti-Tg became positive (anti-TPO 239.2 kIU/L, reference range: <5.6; anti-Tg 7.2 kIU/L, reference range <4.1). Thyroid ultrasonography revealed a heterogeneous background thyroid echogenicity with increase in vascularity, suggestive of diffuse thyroid disease (Figure 1). Her technetium thyroid scan showed findings typical of Graves' disease: diffuse markedly increased uptake over both lobes of thyroid, with associated increased blood flow and increased blood pool on dynamic images (Figure 2). Thyroxine replacement was stopped. She was put on carbimazole and propranolol, with subsequent improvement in the thyroid function (Table 1).

### Literature Review

All the cases reported in the literature regarding thyroid dysfunction after SARS-CoV-2 vaccination are summarized in Table 2. There were 4 cases of Graves' disease after SARS-CoV-2 vaccination, all occurring after mRNA vaccines (age ranged from 28 to 71 years, 3 women and 1 man). In addition, there were 9 cases of subacute thyroiditis reported after SARS-CoV-2 vaccination, which included mRNA, inactivated and adenovirus-vectored vaccines (age ranged from 26 to 67 years, 8 women and 1 man). These cases have been reported in countries in different continents including Asia, Europe, South America and North America.

**Table 2:** Case reports of thyroid dysfunction after SARS-CoV-2 vaccination.

**Sex/Age**	**Country**	**Type of vaccine**	**Past health and family history**	**Time of onset after vaccination**	**TFT at diagnosis**	**Thyroid Ab**	**Thyroid ultrasonography**	**Technetium thyroid scan**	**References**
**Graves' disease**									
F/40	Mexico	mRNA	PH: hypertension, COVID-19 FH: N/A	2 days later	TSH <0.001 mIU/L (N: 0.27–4.4) fT4 3.57 ng/dL (N: 0.93–1.71) fT3 10.5 pg/mL (N: 2.04–4.4)	Anti-TPO +ve Anti-Tg +ve TRAB +ve TSI +ve	Enlargement and hypervascularity	N/A	(8)
F/28	Mexico	mRNA	PH: good FH: N/A	3 days later	TSH <0.001 mIU/L (N: 0.27–4.4) fT4 1.84 ng/dL (N: 0.93–1.71) fT3 9.2 pg/mL (N: 2.04–4.4)	Anti-TPO +ve Anti-Tg -ve TRAB +ve	N/A	Diffuse toxic goiter	(8)
F/71	Austria	mRNA	PH: Graves' disease FH: N/A	56 d after 1st dose 35 d after 2nd dose	fT4 3.56 ng/dL (N: 0.70–1.70) fT3 11.10 pg/mL (N: 2.15–4.12)	TRAB +ve	Multiple confluent anechogenic areas and increased vascularization	Small (partly resected) left lobe and the enlarged right lobe with a patchy inhomogeneous tracer distribution; mildly increased uptake	(9)
M/46	Austria	mRNA	PH: good FH: N/A	15 d after 1st dose	fT4 1.63 ng/dL (N: 0.70–1.70) fT3 5.18 pg/mL (N: 2.15–4.12)	TRAB +ve	Slightly enlarged thyroid; In the hypoechogenic parenchyma, large anechogenic areas with increased vascularization	Patchy, very inhomogeneous Tc99m accumulation; normal uptake	(9)
**Subacute thyroiditis**									
F/57	United States	mRNA	PH: good FH: N/A	35 d after 1st dose 13 d after 2nd dose	TSH <0.008 mIU/L (N: 0.4–4.2) fT4 1.92 ng/dL (N: 0.8–1.5) TT3 137 ng/dL (N: 87–178)	Anti-TPO -ve Anti-Tg -ve TSI -ve	Asymmetrically enlarged hypervascular heterogeneous right thyroid lobe	N/A	(2)
F/49	Germany	mRNA	PH: good FH: father had benign thyroid nodules	14 d after 1st dose	TSH 0.5 mIU/L (N: 0.35–4.94) fT4 9.4 ng/L (N: 7.0–14.8) fT3 3.25 ng/L (N: 1.71–3.71)	Anti-TPO -ve Anti-Tg -ve TRAB -ve	Distinct ill-defined hypoechoic area with decreased blood flow	N/A	(3)
F/35	Turkey	Inactivated	PH: good FH: nil	32 d after 1st dose 4 d after 2nd dose	TSH 0.473 mIU/L (N: 0.38–5.33) fT4 14.1 pmol/L (N: 7.86–14.41) fT3 6.15 pmol/L (N: 3.8–6.0)	Anti-TPO -ve Anti-Tg -ve TRAB -ve	Bilateral focal hypoechoic areas with decreased blood flow	N/A	(4)
F/34	Turkey	Inactivated	PH: mild COVID-19 FH: nil	4 d after 1st dose	TSH 0.01 mIU/L (N: 0.38–5.33) fT4 11.8 pmol/L (N: 7.86–14.41) fT3 5.2 pmol/L (N: 3.8–6.0)	Anti-TPO -ve Anti-Tg -ve TRAB -ve	Bilateral focal hypoechoic areas with decreased blood flow	N/A	(4)
F/37	Turkey	Inactivated	PH: good FH: nil	7 d after 2nd dose	TSH 0.9 mIU/L (N: 0.38–5.33) fT4 13.85 pmol/L (N: 7.86–14.41) fT3 6.05 pmol/L (N: 3.8–6.0)	Anti-TPO -ve Anti-Tg -ve TRAB -ve	Bilateral hypoechoic areas with irregular borders and reduced blood flow	N/A	(4)
M/67	Turkey	Inactivated	PH: controlled hypertension FH: N/A	48 d after 1st dose 20 d after 2nd dose	TSH 0.005 mIU/L (N: 0.27–4.2) fT4 2.87 ng/dL (N: 0.93–1.7) fT3 8.06 pg/mL (N: 2.7–4.3)	Anti-TPO -ve Anti-Tg -ve TRAB -ve	Reduced echogenicity and diffusely heterogeneous texture with pseudonodular areas	N/A	(5)
F/32	Brazil	Inactivated	PH: good FH: N/A	12 h after 2nd dose	TSH 13.2 mIU/L (N: 0.45–4.5) T4 normal	Anti-TPO +ve Anti-Tg +ve	N/A	N/A	(6)
F/26	Germany	Inactivated	PH: good FH: nil	14 d after 1st dose	TSH 1.75 mIU/L (N: 0.35–4.94) fT4 9.3 ng/L (N: 7.0–14.8) fT3 3.72 ng/L (N: 1.71–3.71)	Anti-TPO -ve Anti-Tg -ve TRAB -ve	Distinct ill-defined hypoechoic areas with decreased blood flow	N/A	(3)
F/55	United Kingdom	Adenovirus-vectored	PH: controlled asthma FH: nil	21 d after 1st dose	TSH 0.09 mIU/L (N: 0.3–4.2) fT4 25.2 pmol/L (N: 12.0–22.0)	Anti-TPO -ve	Enlarged thyroid gland with heterogeneous echotexture throughout; no nodules or hypervascularity	N/A	(7)

*F, female; M, male; d, days; PH, past health; FH, family history; TSH, thyroid-stimulating hormone; fT4, free thyroxine; fT3, free triiodothyronine; anti-TPO, anti-thyroid peroxidase; anti-Tg, anti-thyroglobulin; TRAB, TSH receptor antibody; TSI, thyroid stimulating immunoglobulin; N/A, not available; Ab, antibody; TFT, thyroid function test*.

## Discussion

Existing case reports of Graves' disease after SARS-CoV-2 vaccination manifested as the first episode in three patients (8, 9), and as relapse in one patient (9). To our knowledge, our case report represents the fifth case of Graves' disease after SARS-CoV-2 mRNA vaccine in the literature. In contrast to the previous case presentations, the manifestation in our case was a less common presentation—Graves' disease on longstanding background of hypothyroidism requiring thyroxine replacement. The switch from hypothyroid to hyperthyroid state is believed to be stimulated by an external trigger, such as infection, in genetically susceptible individuals (10). Moreover, the development of *de novo* Graves' disease after COVID-19 is believed to take around 6–8 weeks (11). The combination of an uncommon presentation of Graves' disease with a compatible interval after the SARS-CoV-2 vaccination makes the causal relationship even more likely.

Graves' disease has been reported to temporally related to COVID-19, raising the possibility of SARS-CoV-2 in inducing autoimmune thyroid disorders (1). This postulation is supported by the fact that expression of angiotensin-converting enzyme 2 (ACE2), the functional receptor for SARS-CoV-2, is present in many endocrine organs including the thyroid (12). It is of clinical relevance whether SARS-CoV-2 vaccination may be associated with new onset autoimmune thyroid disorders by the same token, and more importantly, as SARS-CoV-2 mRNA vaccines are the first mRNA vaccines in clinical use among human. Molecular mimicry has been proposed to cause thyroid dysfunction after SARS-CoV-2 vaccination. It has been shown that SARS-CoV-2 spike protein, nucleoprotein, and membrane protein all cross-reacted with thyroid peroxidase, and many thyroid peroxidase peptide sequences shared homology or similarity with sequences in various SARS-CoV-2 proteins (13). It is possible that SARS-CoV-2 proteins in the SARS-CoV-2 vaccines cross-react with thyroid target proteins due to molecular mimicry to cause autoimmune thyroid disorders such as Graves' disease. Interestingly, Graves' disease has only been reported among recipients of mRNA vaccines so far, in contrast to the reports of SARS-CoV-2 vaccine-related thyroiditis. The reasons are not entirely clear. As mRNA vaccines, encoding the SARS-CoV-2 spike protein, intrinsically could engage innate immunity that instructs induction of immune protection, the higher reactogenicity of the mRNA vaccine may cause a dysregulated immune system leading to emergence of autoimmune disorders (14, 15). Nonetheless, there could be underreporting of Graves' disease after other types of SARS-CoV-2 vaccines which leads to bias toward the association with mRNA vaccines. Whether family history of autoimmune or thyroid disorders, or genetic factors, may be predisposing factors, is unknown, as family history was not reported in most of these case reports of thyroid dysfunction post-SARS-CoV-2 vaccination, summarized in Table 2.

To date, all the five cases of Graves' disease after SARS-CoV-2 vaccination are not associated with Graves' orbitopathy or thyroid dermopathy. Graves' orbitopathy has been reported to occur in 25% of patients with Graves' disease while thyroid dermopathy in 1.5% of patients with Graves' disease (16). There are factors, such as genetic predisposition or environmental factors, other than anti-TSH receptor antibody levels which contribute to the development of Graves' orbitopathy and thyroid dermopathy (17, 18).

The risk of occurrence of Graves' disease after vaccination has seldom been evaluated. The Vaccine Safety Datalink study evaluated the risk of Graves' disease in recipients of hepatitis B vaccines, a kind of subunit vaccines (19). It did not show an increase in risk of Graves' disease in the ever-recipients, nor was there an association between the time interval between receipt of vaccines and Graves' disease.

In addition to the cases of Graves' disease, nine cases of subacute thyroiditis have been reported in the literature. We have summarized their features in Table 2. Of note, most of the patients presenting with thyroiditis had negative anti-thyroid antibody titers. Interestingly, the types of vaccines involved in these cases of thyroiditis include mRNA, inactivated and adenovirus-vectored SARS-CoV-2 vaccines.

Several mechanisms have been postulated for the occurrence of thyroiditis after SARS-CoV-2 vaccination. The most popular postulation is related to the vaccine adjuvants, contributing to the entity “autoimmune/inflammatory syndrome induced by adjuvants” (ASIA) (20). Post-vaccination phenomena are one of the manifestations in ASIA. Adjuvants are intentionally used to enhance the immunogenicity of vaccines to induce adaptive immune responses. Aluminum salts are commonly used for inactivated vaccines, and is the adjuvant for CoronaVac, the inactivated SARS-CoV-2 vaccine (20). A recent review of post-vaccination ASIA has summarized 50 cases of subacute thyroiditis after vaccination (influenza, human papillomavirus and hepatitis B virus). Indeed, most are anti-thyroid antibody negative except in one case (21). The descriptions are consistent with the existing reports of SARS-CoV-2 vaccine-related subacute thyroiditis. BNT162b2 mRNA vaccine comprises a lipid nanoparticle formulated with a nucleoside RNA encoding a modified SARS-CoV-2 spike protein. The vaccine contains four lipids, of which two are polyethylene glycol (PEG) lipid conjugates that stabilize the lipid nanoparticles and reduce the activity of non-specific binding proteins (22). PEGs may act as an adjuvant and induce an immune response in predisposed individuals, as there are rare reports of reactions to PEGs (23). These probably explain the reports of subacute thyroiditis with SARS-CoV-2 vaccines using different platforms. A similar mechanism has also been postulated to explain the occurrence of Graves' disease post-SARS-CoV-2 vaccination, as it has been shown that human papillomavirus-16/18 AS04-adjuvanted vaccine was associated with an incidence ratio of 3.75 for autoimmune thyroiditis among female (24).

Despite the extensive vaccination against SARS-CoV-2 globally, only a handful of cases of clinically overt thyroid dysfunction have been reported in the literature so far. This could either be related to underreporting or compatible with the rare incidence of post-vaccination adverse events in general. A systematic study of SARS-CoV-2 vaccine recipients regarding their changes in thyroid function and autoantibodies may shed light onto the extent of this problem. Our case report serves to remind clinicians of the potential presentation of thyroid dysfunction after SARS-CoV-2 vaccination.

## Conclusion

Graves' disease can occur after SARS-CoV-2 vaccination. Our case represents the fifth in the literature of Graves' disease after SARS-CoV-2 vaccination, with an unusual presentation on a longstanding history of hypothyroidism. All the five cases reported thus far occurred after mRNA vaccine. Clinicians should remain vigilant about the potential manifestation of Graves' disease after SARS-CoV-2 vaccination in the current COVID-19 pandemic.

## Data Availability Statement

The original contributions presented in the study are included in the article/supplementary material, further inquiries can be directed to the corresponding author/s.

## Ethics Statement

Ethical review and approval was not required for the study on human participants in accordance with the local legislation and institutional requirements. The patients/participants provided their written informed consent to participate in this study.

## Author Contributions

DL and KT performed the literature search and drafted the manuscript. All authors coordinated the patient's care, read, and approved the final manuscript.

## Conflict of Interest

The authors declare that the research was conducted in the absence of any commercial or financial relationships that could be construed as a potential conflict of interest.

## Publisher's Note

All claims expressed in this article are solely those of the authors and do not necessarily represent those of their affiliated organizations, or those of the publisher, the editors and the reviewers. Any product that may be evaluated in this article, or claim that may be made by its manufacturer, is not guaranteed or endorsed by the publisher.
